# Comparative Analysis of Local Versus General Anesthesia and Its Impact on Erectile Dysfunction Risk Following Penile Surgery

**DOI:** 10.7759/cureus.85931

**Published:** 2025-06-13

**Authors:** Marek Harris, Shay Taylor, Elijah McMillan, Samrawit W Zinabu, Noah Wheaton, Da'Jhai Monroe, Sarah Kim, Emmanual Ocampo, Kamdili Ogbutor, Miriam B Michael

**Affiliations:** 1 Urology, Howard University College of Medicine, Washington, DC, USA; 2 Anesthesiology, Howard University Hospital, Washington, DC, USA; 3 Nephrology, Howard University College of Medicine, Washington, DC, USA; 4 Internal Medicine, Howard University Hospital, Washington, DC, USA; 5 Anesthesiology, Howard University College of Medicine, Washington, DC, USA; 6 Otolaryngology, Howard University College of Medicine, Washington, DC, USA; 7 Internal Medicine, Howard University, Washington, DC, USA; 8 Internal Medicine, University of Maryland, Baltimore, USA

**Keywords:** anesthesia, erectile dysfunction, genitourinary trauma, long-term outcomes, sexual function, traumatic penile injury

## Abstract

Background

Traumatic penile injuries (TPIs) pose significant medical and functional challenges, often requiring immediate surgical intervention. The type of anesthesia used during surgery may influence postoperative outcomes, including erectile dysfunction (ED). General anesthesia may impair vasodilation and tissue oxygenation, potentially increasing the risk of ED, while local anesthesia may better preserve neurovascular integrity. However, there is limited research evaluating the relationship between anesthesia type and long-term sexual function in patients with TPI. This retrospective cohort study investigates the impact of local vs. general anesthesia on ED incidence following TPI.

Methods

This retrospective cohort study utilized de-identified electronic health records (EHRs) from the TriNetX database, analyzing 20 years of data. Patients aged 6-24 years who underwent surgery for TPI were categorized into two cohorts: the local anesthesia cohort (n = 512) and the general anesthesia cohort (n = 512). Propensity score matching was used to balance baseline characteristics. The primary outcome was the incidence of ED within one year post-surgery. Statistical analyses included risk ratios (RR), odds ratios (OR), Kaplan-Meier survival estimates, and hazard ratios (HR).

Results

The incidence of ED was 4.7% in the local anesthesia group (24 patients) and 5.1% in the general anesthesia group (26 patients). Risk difference (-0.004, 95% CI: -0.030, 0.022) and RR (0.923, 95% CI: 0.537-1.586) suggested no significant association between anesthesia type and ED risk. Kaplan-Meier survival analysis showed 93.36% of local anesthesia patients and 92.49% of general anesthesia patients remained ED-free at one year (p = 0.750). The HR analysis (HR = 0.914, p = 0.435) further indicated no significant difference in the time to ED development.

Conclusion

This study found no statistically significant difference in the incidence, risk, or timing of ED between local and general anesthesia groups following TPI surgery. These findings suggest that anesthesia type does not independently influence long-term erectile function, providing flexibility in anesthetic selection based on patient and procedural factors. Future prospective studies are warranted to further investigate potential confounders and refine perioperative strategies to optimize sexual function outcomes in TPI patients.

## Introduction

Traumatic penile injuries (TPIs) present complex clinical challenges requiring immediate and specialized intervention [1]. Although genitourinary injuries comprise a small portion of trauma cases, their long-term consequences, especially erectile dysfunction (ED), are significant [1]. Advances in trauma care have improved survival rates, but patients often face profound functional impairments [2]. Notably, the type of anesthesia used during surgery may influence postoperative outcomes, particularly erectile function, yet no standardized anesthetic approach currently exists [1]. Therefore, further exploration is required due to variability in practices and current knowledge gaps that exist in optimizing effective outcomes and quality of life.

In combat-related TPIs, long-term sexual dysfunction remains common due to both physical and psychological factors, such as post-traumatic stress disorder (PTSD). These TPIs are typically associated with long-term structural dysfunctions, extensive rehabilitation steps, and a significant increase in ED cases [2]. Surgical success in these patients depends not only on technique but also on anesthetic management, which can impact neurovascular integrity and hemodynamic stability [3]. The severity and nature of trauma, such as pelvic fractures or penile blunt injuries, further complicate decisions about anesthetic and surgical timing [4,5]. Many factors that improve surgical and postoperative management need to be evaluated to establish a standard for the management of anesthetic tactics chosen during surgery.

The complexities of TPI remain a continuing medical problem due to their serious implications, specifically the possibility of ED. Severe injuries can lead to fibrosis that impairs the recovery and healing of ED and further complicates the situation, increasing the risks of a surgeon's failure and long-term dysfunction [2]. Furthermore, the effects of TPI may lead to more than just mechanical dysfunction, as the trauma invariably affects other vessels and nerves that contribute to the ED in the long run [6]. Hence, there is a need for the development of a comprehensive approach to the surgical procedure for TPIs that ensures resilient healing and long-term quality of life, given the complexities surrounding TPI.

The management of TPI often requires a thorough understanding of the anesthetic impact on surgical outcomes [7]. Emerging literature suggests that local anesthesia may offer benefits in penile surgery, including better neurovascular preservation and a reduced risk of ED [8]. While local anesthesia is commonly used in pediatric and scrotal surgeries, its application in adult penile trauma warrants closer evaluation [9,10]. These are important observations, as the major principles of local anesthesia can be applied to adult patients with similar interventions to achieve higher rates of satisfactory outcomes. The above findings advocate the conscious use of local anesthesia in penile surgery, as it appears to offer safe and effective outcomes similar to scrotal surgeries, with very high procedural success with low complications and high satisfaction rates [11]. However, despite promising results, variability in practice highlights a need for further study.

Local versus general anesthesia in penile surgeries impacts surgical outcomes, particularly the risk of ED [12]. General anesthesia is associated with pathophysiologic disturbances such as vasodilation and reduced tissue oxygenation, both of which increase the likelihood of ED. In contrast, local anesthesia better preserves neurovascular structures, reducing the risk of postoperative complications. Studies, including those by Nasser and Mostafa, suggest that local anesthesia may improve postoperative erectile function by minimizing perioperative neurovascular impairment [13]. However, clinical practices vary widely, revealing a lack of standardized anesthetic guidelines and underscoring the need for further research on optimal anesthetic methods in penile surgery.

Encouraging findings on the use of local anesthesia for penile fractures highlight its potential to reduce complications and accelerate recovery [13,14]. This approach supports earlier return to sexual activity and preservation of erectile function through superior neurovascular protection. Fewer intraoperative complications may correlate with reduced ED risk, suggesting that future research and guideline development should prioritize anesthetic technique as a modifiable factor for improved outcomes.

General anesthesia increases ED risk by reducing perfusion to penile tissues through systemic vasodilation, lowering blood pressure, and impairing oxygen and nutrient delivery [2,15]. It may also contribute to venous pooling and vascular dysfunction. Despite these concerns, it remains a commonly used treatment, although it is linked with greater hemodynamic instability and potential ED [16]. Given these effects, anesthetic choice must be carefully considered, especially in patients at risk for ED. Local anesthesia, by contrast, offers neurovascular protection with minimal systemic impact. It selectively blocks targeted nerves and vessels, maintaining hemodynamic stability and preserving erectile structure [14,17]. Its localized action may also enhance wound healing and minimize vascular compromise, ultimately improving recovery and lowering ED risk. Additionally, it reduces overall physiological stress, further supporting favorable outcomes.

The impact of anesthesia type on erectile outcomes remains an important focus of study. While retrospective data show mixed results, prospective studies, such as those by Bal et al. [11], highlight the benefits of local anesthesia in reducing complications and improving recovery. These discrepancies point to a critical need for more robust research. As anesthetic selection plays a key role in both short- and long-term genitourinary function, this study addresses a notable gap in urologic trauma literature. Clarifying the influence of anesthetic methods will help guide perioperative care, ultimately enhancing patient recovery, sexual function, and overall quality of life. Such insights are vital, given the psychological and functional ramifications of ED on patients suffering from TPI.

## Materials and methods

Study design

This study employed a nationwide retrospective cohort study. This investigated the incidence of ED in patients who had a TPI and underwent surgery using local anesthesia compared to those having surgery with general anesthesia. De-identified electronic health records (EHRs) from the TriNetX database, encompassing 20 years of data, were analyzed. TriNetX is a global federated health research network that provides access to electronic medical records, including diagnoses, procedures, medications, laboratory values, and genomic information, from 93 healthcare organizations (HCOs) grouped into a network called Research using Natural Language Processing (NLP). The analysis process consisted of two main steps:

Defining the Cohorts

Patients were categorized based on specific query criteria into two cohorts. Local anesthesia cohort (Cohort 1, n = 512): patients between the ages of six and 24 years with TPI who underwent surgery using local anesthesia within one year before September 27, 2024. General anesthesia cohort (Cohort 2, n = 512): patients between the ages of six and 24 years with TPI who underwent surgery using general anesthesia within one year before September 27, 2024.

Setting Up and Running the Analysis

This involved defining the index event, the time frame, and outcome criteria. The index event is defined as the point in time when each patient enters the analysis, specifically when the patient had surgery for TPI. The time window is defined by analyzing the outcomes from the same day as the index event and continuing for one year. Outcome refers to the primary outcome of interest, which was the occurrence of any ED within one year after being included in the study population. The ICD-10 code of ED refers to the specific code indicating the diagnosis of ED for reimbursement purposes, where ED is defined as a disorder characterized by persistent or recurrent inability to achieve or to maintain an erection.

Cohort construction

The initial cohort consisted of 131,629,360 enrolled participants. After applying the inclusion and exclusion criteria, the final study population was divided into two groups. The exposed cohort (Cohort 1) consisted of patients who underwent documented penile injury surgery with local anesthesia. This query was run on the network Research, with 93 HCOs queried, and a total of 65 providers responded with patients. The comparison cohort (Cohort 2) included patients with documented penile injury surgery with general anesthesia. This query was run on the network Research, with 93 HCOs queried, and a total of 82 providers responded with patients.

Inclusion and exclusion criteria

Inclusion Criteria

The study encompassed individuals aged 6-24 years and diagnosed with TPI based on the specified ICD-10 codes. Patients determined to be in either the local or general anesthesia cohorts were placed based on their ICD-10 indication. The age range of six to 24 years was chosen to exclude other potential variables that could influence the presentation of ED.

Exclusion Criteria

The study excluded individuals under the age of six years and above 24 years and those with known pre-existing ED. Additional exclusions related to the biggest risk factors for ED, such as peripheral vascular disease and diabetes, were conducted.

Data analysis

Statistical analysis was performed using the TrinetX platform. Propensity score matching was employed to balance baseline characteristics between the two cohorts. The matched characteristics were age at index, sex, peripheral vascular disease, and diabetes. Measures of association analysis were utilized to see the risk of illnesses between the groups. The number of outcome events that occurred within the time window in each group was described, and the mean number of outcome events was calculated for those with outcomes during the study period using a number of instances analysis. The number of instances was grouped by visit, which counts any visit for the outcome as one, regardless of the number of times it occurred. Additionally, Kaplan-Meier estimates were used to assess the development of ED probability at the end of the study period, and hazard ratios (HR) were calculated using the same analysis.

Ethical considerations

This retrospective study is exempt from informed consent. The data reviewed is a secondary analysis of existing data, does not involve intervention or interaction with human subjects, and is de-identified according to the de-identification standard defined in the HIPAA Privacy Rule.

## Results

Cohort

Two cohorts of patients are being studied: (1) Local Anesthesia (Local): 512 patients treated with local anesthesia. (2) General Anesthesia (General): 512 patients treated with general anesthesia. Patients were matched for age, with 512 patients in each group.

Outcome

A total of 24 patients experienced ED following the administration of local anesthesia, whereas 26 patients experienced ED following the administration of general anesthesia.

Risk

The proportion of patients who experienced ED within each group. The risk of experiencing ED with local anesthesia was 0.047 (4.7%), whereas with general anesthesia, the risk of ED was 0.051 (5.1%) (Table 1).

**Table 1:** Risk of Erectile Dysfunction

Cohort	Total Patients #	Erectile Dysfunction (Outcome)	Risk	Risk Difference	P-value of Risk Difference	Risk Ratio	Odds Ratio	Kaplan-Meier Survival Probability (%)
Local	512	24	0.047	-0.004	0.772	0.923	0.919	93.36%
General	512	26	0.051	-	-	-	-	92.49%

Risk difference

The difference in risk between the two groups is -0.004, while the difference in ED risk between local and general anesthesia is small (approximately 0.4% lower in the local anesthesia group) (95% CI: -0.030, 0.022). The difference could fall between a 3% decrease and a 2.2% increase. The z- and p-values are determined to be -0.290 and 0.772, respectively. There is no strong evidence that the two groups differ in risk.

Risk ratio (RR)

The risk of ED between the two groups is examined and was found to have an RR of 0.923, indicating that patients with local anesthesia exhibit approximately 92.3% of the risk of developing ED compared to those with general anesthesia. The 95% CI (0.537 to 1.586) is not statistically significant.

Odds ratio (OR)

The odds of developing ED are 0.919, indicating that the odds of ED are slightly lower in the local anesthesia group; however, the confidence interval (0.520 to 1.624) is not statistically significant.

Kaplan-Meier survival analysis

The percentage of patients who did not develop ED by the end of the study period was assessed. It was found that with the local anesthesia group, 93.36% of patients did not develop ED, whereas in the general anesthesia group, 92.49% of patients did not develop ED.

Log-rank test

The χ² (chi-square) value is 0.102, with 1 degree of freedom and a p-value of 0.750. There is no statistically significant difference in the likelihood of not developing ED between the two anesthesia groups.

Hazard ratio

The risk of developing ED over time was examined in both groups. The HR is 0.914 for patients undergoing local anesthesia, who have a slightly lower risk of developing ED over time compared to those with general anesthesia. However, it is not statistically significant. The χ² value is 0.610, with 1 degree of freedom and a p-value of 0.435, showing no significant difference between the two groups.

Summary

The analysis indicates that there is no statistically significant difference between local and general anesthesia in terms of the risk, odds, or timing of ED following surgery. The p-values in both the risk analysis and Kaplan-Meier survival analysis indicate that any observed differences between the groups could be due to chance rather than a real effect of the anesthetic method used.

## Discussion

In this study, we examined two cohorts of patients who underwent penile surgery to assess the impact of anesthesia type on the incidence and risk of ED. The two cohorts, one receiving local anesthesia (n = 512) and the other receiving general anesthesia (n = 512), were matched for age to reduce confounding factors related to this known risk factor for ED. The outcomes of interest were the incidence and risk of ED following surgery.

The data show that the incidence of ED was similar in both anesthesia groups, with 24 patients (4.7%) in the local anesthesia group and 26 patients (5.1%) in the general anesthesia group experiencing ED. This translates to a risk difference of -0.004 (95% CI: -0.030, 0.022), indicating a small 0.4% lower risk of ED in the local anesthesia group. However, this difference is not statistically significant, as evidenced by the wide confidence interval, which ranges from a 3% decrease to a 2.2% increase in risk. The z-value of -0.290 and the p-value of 0.772 also suggest that there is no strong evidence to support a difference in ED risk between the two anesthesia types.

The RR between the two groups was calculated to be 0.923 (95% CI: 0.537 to 1.586), indicating that patients who received local anesthesia had approximately 92.3% of the risk of developing ED compared to those who received general anesthesia. Similarly, the OR was 0.919 (95% CI: 0.520 to 1.624), suggesting slightly lower odds of ED in the local anesthesia group. Neither the RR nor the OR was statistically significant. This suggests that the type of anesthesia does not appear to play a critical role in influencing the odds or risk of postoperative ED.

In our study, we used Kaplan-Meier survival analysis to evaluate the time needed to develop ED in both anesthesia groups. At the end of the study period, 93.36% of patients in the local anesthesia group and 92.49% of patients in the general anesthesia group did not develop ED. There was no statistically significant difference between the two groups in terms of the likelihood of not developing ED. The log-rank test (χ² = 0.102, p = 0.750) was confirmed.

The HR for developing ED over time was 0.914 (χ² = 0.610, p = 0.435), indicating a slightly lower risk of developing ED over time in the local anesthesia group compared to the general anesthesia group. However, this difference was not statistically significant, further supporting the conclusion that the type of anesthesia does not significantly affect the risk of ED over time.

Given the lack of statistically significant differences in the risk, odds, or timing of ED between the two anesthesia groups, this study suggests that the choice of anesthesia, local versus general, may not be a critical factor in determining postoperative erectile function. This finding is clinically important, as it allows surgeons and anesthesiologists to prioritize other considerations, such as patient preference, comorbidities, and procedural requirements, when deciding between local and general anesthesia for penile surgery.

The findings of this study underscore the complexity of factors influencing erectile function post-trauma surgery. While our results did not demonstrate a significant difference in ED outcomes between anesthesia types, they highlight the need for a holistic approach to patient management in TPIs. The absence of significant differences suggests that surgeons have the flexibility to choose the type of anesthesia based on other patient-specific factors without worrying about long-term ED.

Moreover, the role of anesthesia in preserving erectile function is an important area for future research. Detailed studies incorporating a larger sample size, varied injury severities, and different surgical techniques could provide deeper insights into the optimal perioperative strategies. Such research could pave the way for tailored anesthesia protocols that minimize the risk of ED and improve overall recovery.

This paper contributes to the broader understanding of postoperative sexual function, emphasizing the importance of personalized medicine in urologic trauma care. It also sets the stage for future studies to explore innovative anesthesia techniques and their long-term effects on sexual health. By refining our understanding of these dynamics, healthcare providers can better support the recovery and well-being of individuals undergoing surgery for TPIs.

Limitations

The findings do not demonstrate a clear advantage of one type of anesthesia over the other, but the study's limitations should be acknowledged. The study did not control for other potential risk factors for ED, such as the extent of penile injury, the duration of the surgery, or preexisting vascular or neurological conditions. Future research could address these variables and provide more granular insights into the impact of anesthesia on erectile function following penile surgery.

A retrospective cohort study design has several drawbacks. With the study relying on pre-existing data, the patient selection for local or general anesthesia could be based on comorbidities and patient risk factors that could influence outcomes, and the groups may differ in ways that are not accounted for. The analysis relied on historical data, which may be inaccurate or incomplete, and the nature of the penile injury or presence of comorbidities was not accounted for. Although we have removed patients with pre-existing ED, there is still a risk of incomplete or erroneous information, which could compromise the validity of the results. Although we have the temporal order of exposure to anesthesia and then subsequent development of ED, we have not evaluated or controlled for the exact timing and progression to ED, which limits our ability to establish causal relationships. Since this was an observational study, we did not randomize the patients, and the differences between the general anesthesia group and the local anesthesia group may not be distributed equally, and causality cannot be firmly established. We have attempted to increase the generalizability of these results to the United States population by using a large base population of 164 million from which to draw our patients.

## Conclusions

In conclusion, this cohort study found no statistically significant difference in the incidence, risk, or timing of ED between patients who received local anesthesia and those who received general anesthesia for penile surgery. These results suggest that anesthesia type alone does not significantly influence postoperative erectile function, offering flexibility in anesthetic choice without increasing the risk of ED. Further research with more comprehensive control of confounding variables is necessary to confirm these findings and explore potential mechanisms underlying anesthesia-related outcomes in penile surgery.

While retrospective cohort studies are valuable for exploring associations, they are inherently limited by selection bias, incomplete data, confounding, and the inability to definitively establish causality. These limitations highlight the need for future prospective studies or randomized controlled trials to more definitively assess the relationship between anesthesia types and the risk of ED following penile surgery.
